# The Effects of High-Intensity Interval Training (HIIT) on Fall Risk Factors in Healthy Older Adults: A Systematic Review

**DOI:** 10.3390/ijerph182211809

**Published:** 2021-11-11

**Authors:** Michal Elboim-Gabyzon, Roie Buxbaum, Roei Klein

**Affiliations:** Department of Physical Therapy, Faculty of Social Welfare & Health Sciences, University of Haifa, Haifa 3498838, Israel; roiebuxbaum123@gmail.com (R.B.); Roeiklein1@gmail.com (R.K.)

**Keywords:** high-intensity interval training approach, HIIT, falls risk, balance, older adults

## Abstract

High-intensity interval training (HIIT) improves functional capacity, muscle power and physical performance in older adults with and without comorbidities. The aim of this study was to explore the effectiveness of HIIT as a method for reducing major fall risk factors (balance, muscle strength and physical activity) in older adults. A systematic literature search was conducted following the PRISMA guidelines. A computerized search was conducted using electronic databases (PubMed, CINAHL, Cochrane Library, APA PsycInfo, Web of Science, Scopus, PEDro, and AgeLine) published up to July 2021. Eleven papers (9 studies) of moderate quality (mean of 5.5 in Pedro scale) involving 328 healthy older adults met the inclusion criteria. Studies were characterized by high heterogeneity in terms of methodology, HIIT modality and protocol, subject characteristics, and outcome measures. Results indicate that HIIT cannot be recommended as a single modality for fall prevention in older adults due to insufficient data and no consensus among the studies. HIIT appears to be a safe and well-tolerated supplement to proven fall prevention programs, due to its effects on lower limb strength reflected in functional performance tests, and on dynamic balance and subjective balance perception. However, caution is warranted following HIIT, especially after the first session, due to possible temporary instability.

## 1. Introduction

Falls are a major global public health challenge, causing a substantial and increasing health and economic burden on older adults and society at large [1,2,3]. The World Health Organization (WHO) developed a model that categorizes fall risk factors into four dimensions: biological, socioeconomic, behavioral, and environmental [1]. Of these, behavioral factors have the highest impact on fall frequency [1]. Accordingly, lifestyle behavioral changes, such as increasing physical activity, have the potential to prevent falls [4]. 

According to the WHO guidelines, older adults should engage in the general program recommended to adults (18–64 years old). Such a program should include at least 150–300 min of moderate-intensity aerobic exercise, or at least 75–150 min of vigorous intensity aerobic exercise per week (or an equivalent combination of the two). Furthermore, it is recommended that they participate in muscle-strengthening activities at moderate or greater intensity that involve all major muscle groups, on 2 or more days per week. For older adults, the recommendation is that they add to this program, on three or more days per week, multicomponent physical activities at moderate or greater intensity, in order to enhance functional capacity and prevent falls [5]. Most older adults do not follow these guidelines and maintain a sedentary lifestyle, despite the well-established positive benefits of a physically active lifestyle and the detailed guidelines provided [4,6,7].

Time constraints, lack of motivation or interest, and the perception of exercise regimes as boring are among the most frequent barriers to physical activity in older adults [8,9]. These barriers must be considered when exercise interventions are prescribed [4,6]. One type of exercise that may bypass these barriers, particularly time constraints, is the high-intensity interval training (HIIT) approach. This regime involves short to long bursts/bouts of highly intensive exercise/activity interspersed with periods of recovery that consist of low-intensity exercise or rest with various work-to-rest ratios [10].

HIIT is a time-efficient alternative strategy to moderate-intensity continuous exercise training (MICT) [11]. A typical HIIT session may be up to three times shorter than a traditional MICT session [12]. However, studies have reported health benefits and physiological adaptations similar to MICT in a much shorter timeframe, particularly for untrained persons [10]. HIIT also enables spending longer training time at high targeted intensities of >90% VO_2_max and maximal heart rate, referred to as the “red zone” [13].

HIIT has been prevalent among athletes for many years [14,15,16]. In recent years, HIIT protocols adjusted for non-athletic, healthy older adults, and adults with morbidities such as cardiac disease and rheumatoid arthritis have emerged [17,18,19,20,21]. This exercise approach has several advantages. In addition to being time saving compared with continuous aerobic training, HIIT has been shown to have advantages in terms of cardiorespiratory aerobic capacity (e.g., increased maximal oxygen consumption). This phenomenon is attributed to increases in the heart’s pumping capacity and in the mitochondrial activity [22,23]. Increased aerobic capacity is related to health benefits such as longer lifespan, functional independence, psychological well-being, and quality of life [24,25]. HIIT has also been proven to have a positive effect on metabolic outcomes, such as improving blood glucose control and increased utilization of glucose and lipids [20,26,27]. 

HIIT has been shown to improve functional capacity, muscle power, and physical performance in older individuals with and without comorbidities such as heart failure and diabetes mellitus [27,28,29]. While HIIT programs are regarded as more enjoyable and motivating than standard continuous exercise programs, they have also been shown to be safe for older adults with and without heart disease [19,30,31,32].

These findings, in particular the positive effects of HIIT on muscle power [27], justify the examination of HIIT as a strategy for preventing falls by lowering intrinsic risk factors such as muscle strength and power [3,33,34,35,36]. However, to the best of our knowledge, a comprehensive review of the literature, including a summary and analysis of existing studies on this issue, has not been conducted to date. In order to fill this gap, the purpose of the present study was to answer the question: Is HIIT an effective method for reducing major fall risk factors (balance, muscle strength and physical activity) in older adults? An additional objective was to draw practical applications for the use of HIIT as a fall prevention modality in healthy older adults living in the community.

## 2. Methods

### 2.1. Data Sources and Literature Searching

A systematic literature search was conducted following the PRISMA guidelines for systematic reviews and meta-analyses [37]. The study was registered as health and social care (PROSPERO: CRD42020173149). The review protocol is available on the PROSPERO website (https://www.crd.york.ac.uk/prospero/display_record.php?RecordID=173149, accessed on 5 July 2021). 

A computerized search was conducted using electronic databases for literature published up to July 2021. The search dates were from March 2020 to July 2021. The following electronic databases were searched using identical search strings and the MeSH (medical subject headings) terms specific to the selected database:

- PubMed
- CINAHL
- The Cochrane Library
- APA PsycInfo
- Web of Science
- Scopus
- PEDro
- AgeLine

In addition, a gray literature search was conducted using ClinicalTrials.gov and Google Scholar. Conference abstracts, dissertations, theses, and articles published in non-peer-reviewed journals were not included. The search strings were further limited to original research studies published in peer-reviewed journals written in English with no restrictions on the publication year. We were assisted by a librarian in formulating the database search strategy.

### 2.2. Inclusion and Exclusion Criteria

Clinical trials for which full texts were available were included. These included randomized control trials (RCTs), cohort studies (prospective observational studies), and cross-over studies. Single case studies were excluded. 

Eligibility criteria were determined according to the PICO framework, that is, Participants/Population/Problem, Intervention, Control or Comparison, and Outcome [38]:

- Participants/Population/Problem: Clinical trials were conducted on humans and healthy older adults with an average age of 60 and over for both genders. Studies with comorbidities such as obesity, metabolic syndrome, neurological disorders, cardiovascular disorders, diabetes mellitus, or pulmonary disease were excluded.
- Intervention: The intervention protocol included at least one group performing an intervention of HIIT, defined as high-intensity exercise separated by periods of low to moderate-intensity or rest. High-intensity was defined as 90–95% of peak heart rate, 90% of maximal oxygen uptake, at least 75% of peak work rate, or perceived exertion (RPE) of at least 16 on the Börg scale [39]. Only studies in which it was possible to isolate the effect of HIIT were included. For example, if a food supplement was provided only to the HIIT group, the study was excluded. In addition, no restrictions were included in the treatment characteristics, such as duration of protocol, frequency of intervention, number of interventions, or type of HITT modality (e.g., walking/running, cycling).
- Comparison/Control: Treatment was compared to control groups either receiving no treatment or an alternative treatment including another training/exercise modality.
- Outcome measures: Outcome measures assessed at least one of the following: balance, stability, fall frequency, lower extremity muscle strength and/or power, gait performance, physical activity or quality of life.

### 2.3. Study Selection, Data Extraction, and Quality Assessment

Two of the authors independently searched the identified databases and reviewed titles and abstracts in accordance with the criteria described above. The names of manuscript authors and their institutions, as well as the names of the journals, were identified by the reviewing authors. To ensure that all relevant articles were included, if the abstract or title did not provide sufficient details regarding the exercise protocol, the methods section of the manuscript was read. In addition, reference lists of the identified articles were manually examined for additional relevant titles. Subsequently, full-text articles considered potentially applicable were reviewed by the two authors for eligibility.

Covidence software (https://www.covidence.org/, accessed on 1 July 2021) was used to manage the literature review and remove duplicates. The two authors who conducted the search in the previous stage performed data extraction independently. A structured form inserted into the Covidence software program to reduce the possibility of data entry errors was used. A third author then reviewed all tables to ensure accuracy. Differences in the data details were settled by mutual agreement. In cases of disagreement, consensus was achieved through discussion with a third reviewer. Whenever necessary, the first or corresponding author of the manuscript was contacted to obtain the relevant missing data.

The following data were extracted from each eligible study: general study information (authors’ last name, publication year, study design, study aim, number of participants, and outcome measures), participants information (average age, gender, baseline activity level), and intervention data for HIIT (description, definition of high-intensity, supervision, location, number of participants, number of bouts, work intensity, work time, rest interval type and duration, and total intervention duration), information regarding the control group/s (number of participants in each group, type of control—passive or modality if there was a modality group—the components, work intensity, duration, follow-up period, and dropout rate), and outcomes (times of measurement, effect). In addition, the number and types of adverse events were extracted. The outcome measure data were provided by mean values and measures of variability, either published or obtained from the authors. We contacted the corresponding author via email in the following cases: when two or more studies were published by the same authors in order to confirm no double-counting of patients, and in cases where the mean values and measures of variability were presented only in figures. 

With regard to risk of bias (quality) assessment, two of the authors independently assessed the methodological quality of the included studies using the Physiotherapy Evidence Database (PEDro) scale [40,41], which assesses the external and internal validity and statistical soundness of studies included in systematic reviews. Although the PEDro scale includes 11 items (eligibility criteria, random allocation, concealed allocation, baseline comparability, blind subjects, blind therapists, blind assessors, adequate follow-up, intention-to-treat analysis, between-group comparisons, point estimates, and variability), the total score ranges between zero and 10, as the first item (eligibility criteria) does not add to the total score. Higher scores indicate higher quality. The quality of scores on the PEDro scale were interpreted as follows: a total PEDro score equal or higher than 6 was considered high quality, a score of 4 or 5 was viewed as moderate quality, and a score of 3 or less was considered low quality [41].

Articles not yet appearing in the PEDro database (if any) were scored by two of the authors, with disagreements between them resolved by discussion, with input from a third reviewer if necessary.

## 3. Results

**Figure 1:**
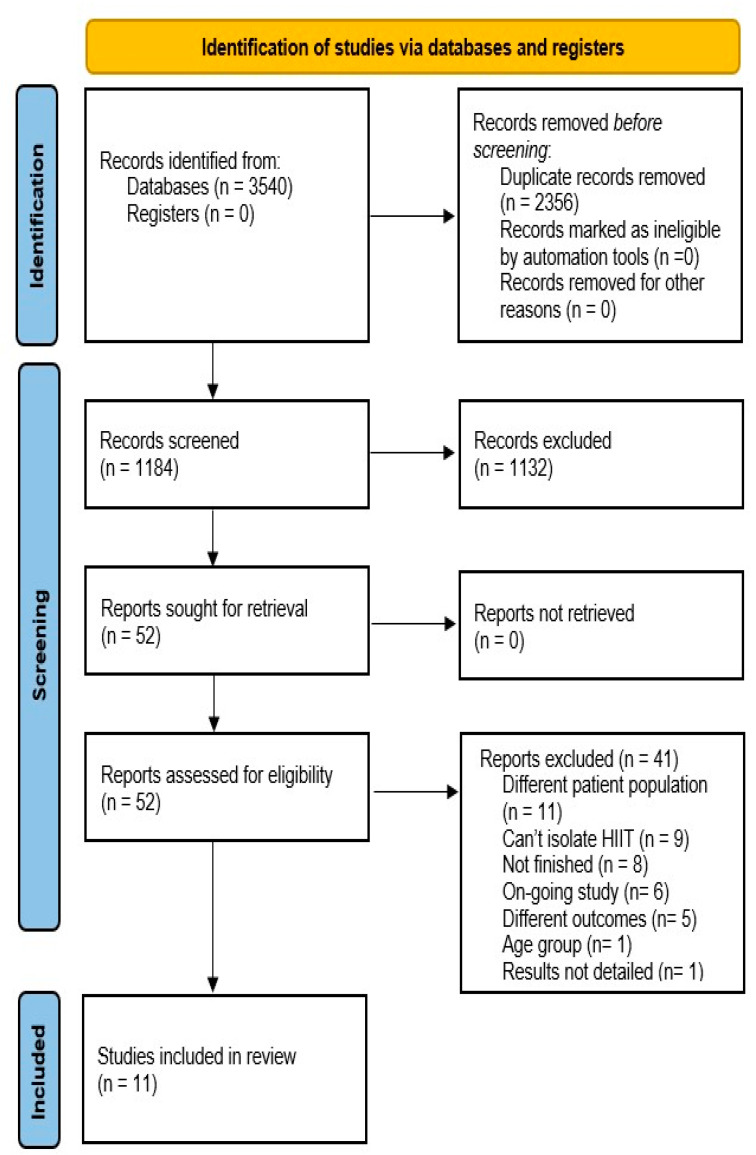
PRISMA flow diagram to depict search strategy results.

An initial literature search of all the included databases resulted in 3540 references. After 2356 duplicates were removed, 1184 titles and abstracts were screened for eligibility. A total of 1173 studies were excluded for reasons detailed in Figure 1. A total of 11 studies met the inclusion criteria and were included in this systematic review [3,33,35,42,43,44,45,46,47,48,49]. It should be noted that three pairs of studies were based on the same population samples [3,33,43,44,47,49].

### 3.1. Overview and General Characteristics of the Analyzed Studies

The 11 studies included in this review [3,33,35,42,43,44,45,46,47,48,49] were published between 2015 and 2020. Of these, seven were parallel RCTs [3,35,42,46,48,49]. One study used a randomized control crossover design with two age groups of 20 healthy older adults (age: *M* = 70, *SD* = 3.8) and 20 young adults (age: *M =* 27.1, *SD =* 3) [33], and additional studies were conducted on the same sample with a pre-post design [47]. Two studies used a within-subject design [43,44].

The characteristics of the participants involved in the 11 included studies is detailed in Table 1. The total number of older adults across all the studies was 328; focusing only on the HIIT intervention, the number of participants was 143. Three studies reported the age range of the sample [35,46,48], while the other studies reported an average age (*M*) and standard deviation (*SD*) [3,33,35,42,43,44,45,46,47,48,49].

In the three studies that reported age range [35,46,48], the minimum age was 50 and the maximum age was 81 [48]. The average age of the rest of the sample ranged between 67.7 [42] and 70 [33,47]. With regard to the HIIT group, the average age ranged from 61.9 [48] to 70 [33,47].

Regarding the gender of the participants, four papers included only males [35,43,44,45], six papers included both genders [3,33,46,47,48,49], and one study included only females [42].

None of the studies reported previous history of falling.

Of the 328 participants distributed over the exercise groups in the nine studies (11 papers), 143 participants completed a form of HIIT (see Table 2).

Eighty-nine participants completed another exercise modality: resistance exercise, 22 subjects [46]; moderate continuous aerobic training, 43 subjects [42,45,46]; moderate-intensity interval training, 24 subjects [3,49] (see Table 1 for details). A total of 89 participants did not exercise at all (control group) [3,35,42,46,48,49].

All the subjects were healthy older adults as per the inclusion criteria. The pre-intervention level of activity was reported in most of the papers [33,35,43,44,45,46,47,48] (see Table 1 for details). Although in some studies the level of activity was assessed accurately by means of validated questionnaires [33,43,44,47], in two studies, the information was collected by self-report with no mention of any specific questionnaire [35,48], and in one study it is not clear how this information was gathered [46]. Furthermore, three papers did not report the prior activity levels of the participants [3,42,49]. No uniformity was found between the articles in terms of the participants’ pre-intervention activity level which ranged from sedentary lifestyle (inactive) [35,46,48] to physically active [33,47] and moderately active [43,44,45] (see Table 1).

### 3.2. Protocols and Periodization of HIIT Interventions

The 11 reviewed studies [3,33,35,42,43,44,45,46,47,48,49] utilized various forms of HIIT that varied in modality, intensity, work-to-rest ratio, intervention duration, intervention frequency, and total number of interventions, as presented in Table 2.

Four of the 11 studies conducted HIIT using stationary cycle ergometers with various levels of resistance [35,43,44,45], three evaluated treadmill walking at different speeds and levels of inclination [33,46,47], and four performed multiple forms of strength training with and without external resistance [3,42,48,49].

The intensity of HIIT training was measured in one of four ways: VO_2_ max, maximal heart rate, heart rate reserve, and rate of perceived exertion using the Börg scale. An intensity of 85–95% of VO_2_ max was applied in three studies [43,44,45], 90–95% maximal heart rate was used in six studies [3,33,46,47,48,49], a heart rate reserve of greater than 90% as measured indirectly by estimating the peak power of the lower leg muscles was used in one study [36], and a score of 16–18 of perceived exertion according to the Börg scale in one study [42].

The number of bouts in the included studies ranged from a minimum of four bouts [3,33,46,47,48,49] to a maximum of 12 bouts [42]. In six of the studies there were four bouts of high-intensity [3,33,46,47,48,49], in three studies there were seven high-intensity bouts [43,44,45], in one study there were six high-intensity bouts [36] and in one study, there were 12 high-intensity bouts [42]. 

High volume protocols of HIIT were used in six of the studies: five studies applied a 4 × 4 protocol [3,33,46,47,49], and one study used a protocol of 12 × 1.5 [42]. Low HIIT volume was used in five studies, with each one of the five including different combinations of number of bouts and duration of the activity interval: 7 × 2 [43,44,45], 6 × 0.5 [35], and 4 × 1.25 [48]. The duration of the work interval ranged from 30 s [36] to 4 min [3,33,46,47,49]. One study applied 1–1.5 min of work [42], one study used 1.25 min of work [48], three studies applied 2 min of work [43,44,45], while the other five studies applied 4 min of work [3,33,46,47,49].

Periods of passive recovery [48] or active recovery were interspersed between work bouts in 10 out of the 11 studies [3,33,35,42,43,44,45,46,47,49]. The intensity of the active recovery was assessed in the same four ways as the intensity of the training: VO_2_ max, maximum heart rate, heart rate reserve, and rate of perceived exertion using the Börg scale. Regarding the intensity of the rest recovery, 40% of VO_2_ max was applied in three studies [43,44,45], 70% maximal heart rate was applied in three studies [33,46,47], 50–70% HR max was applied in two studies [3,49], a score of 10–11 of perceived exertion according to the Börg scale was applied in one study [42], and in one study the intensity of active rest was not reported [35]. The recovery duration ranged from a minimum of 2 min [43,44,45] to a maximum of 3 min [3,33,35,46,47,48,49], with one study reporting a value of 2–2.5 min rest duration [42].

The total number of HIIT sessions ranged from one [33,47] to 48 [46], which lasted between 6 [36] to 18 weeks [42], and at a training frequency ranging between one [36] and three [46] sessions per week [36,46].

### 3.3. Protocols for Additional Intervention Groups and Control Groups

Details of the intervention group compared with the HIIT group or/and with the control group are presented in Table 3.

In some studies, the effect of HIIT was compared to that of other types of exercise, as shown in Table 2 [3,33,42,43,44,45,46,49]. Two of these studies were not RCTs, but were within-subject designs [43,44]. The other exercise modalities included: (1) moderate-intensity continuous treadmill walking was performed in two studies [33,46]; (2) moderate-intensity continuous cycling or treadmill walking in one study [45]; (3) resistance training in four papers [42,43,44,46]; and (4) moderate-intensity interval training of TRX [3,49]. One study used a pre-post design and compared an older population to a younger population [47], and five studies included control groups that did not undergo exercise training [3,35,42,46,48,49].

### 3.4. Preconditioning and Familiarization

Five of the 11 studies included a familiarization period prior to the exercise intervention, during which subjects preformed a similar or simplified version of the training in order to familiarize themselves with the exercises [3,35,42,44,49]. No consistency was found between the reviewed studies in terms of the duration of the preconditioning period which ranged from two weeks [42], four weeks [3,49], and six weeks [35]; in one study, this information was not provided [43]. Four studies conducted familiarization of the physical tests and outcome measures before data were collected [33,36,43,44], while several other studies did not provide data regarding preconditioning and familiarization [45,47,48,50].

### 3.5. Supervision and Monitoring of the Treatment Intensity

Seven of the 11 studies under review reported that the participants were supervised during the HIIT [3,35,42,44,45,48,49]. Of these, only three reported who performed the supervision [3,42,49].

The intensity of the training and the recovery interval was monitored by means of continuous heart rate monitoring during training in seven studies [3,33,35,46,47,48,49]. Three studies monitored the intensity of the training and recovery by measuring the VO_2_ max during the warm-ups and during the last minute of each training load [43,44,45]. One study [42] monitored the required intensity by participants’ self-reported rating of perceived exertion after each block of work and rest of the HIIT.

### 3.6. Location of the Intervention

All 11 of the reviewed studies were conducted in a laboratory setting [3,33,35,42,43,44,45,46,47,48,49].

### 3.7. Adherence

The number of participants who dropped out and the training attendance percentages are detailed in Table 4. The attendance rates of the study participants were not reported in three studies [33,46,47]. Six studies reported the general attendance rates of all participants but no specifics for each group [3,35,43,44,45,49]. Two studies reported attendance rates of 84% [42] and 99% [48] for the HIIT groups. 

### 3.8. Adverse Effects

Seven studies reported that no adverse effects occurred during HIIT training [3,35,42,44,45,46,48]. Four studies did not state whether adverse effects occurred [33,43,47,49]. One study [46] reported that five participants dropped out of the study due to adverse effects not related to the study or to HIIT (the five adverse events: MICT = 4, CON = 1, including eye surgery, foot surgery, clavicle fracture, and two hip fractures after a fall) [42]. One study reported that two participants dropped out of the HIIT group as a result of injury which was unrelated to the study. Finally, one study [48] reported that one participant missed two HIIT sessions because of an injury unrelated to the study.

### 3.9. Comparison between HIIT and Other Exercise Modality or Non exercise

#### 3.9.1. Outcome Measure and Intervention Effect

Details are presented in Table 5.

The effect of HIIT and other exercise modalities or nonexercise in the included studies was examined using a number of outcome measures which can be divided into four categories:

1. Muscle function and morphology. This category can be further divided into the following subcategories: (1a) upper limb strength and power [42,48,49] and (1b) lower limb strength, muscle area, volume, and activity [33,35,42,43,44,47,48];
2. Balance and subjective balance perception [3,33,35,42,47];
3. Gait and level of physical activity [3,42,45,49];
4. Quality of life [48,49].

(1) Muscle function and morphology

(1a) Upper limb strength and power.

Three studies evaluated upper limb strength [42,48,49]. The arm curl test was presented in one study [42], whereas hand grip strength was shown in three studies [42,48,49].

One study showed that HIIT was more beneficial in improving the arm curl test than MICT and the non-exercise group [42].

With regard to the effect of HIIT on hand grip strength, one study demonstrated an increase in hand grip strength; however, this improvement was equal to MIIT and CON [49]. In contrast, one study stated that HIIT caused a “likely trivial effect” on the dominant handgrip strength and “possible small beneficial effects” on non-dominant handgrip strength [48]. In the third study [49], HIIT did not have an effect on hand grip strength.

(1b) Lower limb strength, muscle area, volume and activity.

Lower limb strength, muscle mass, and muscle activity was measured in seven studies [33,35,42,43,44,47,48].

Three studies evaluated the strength of the lower limb muscles without targeting specific muscles. One study used the 30 s sit-to-stand test as a measurement of functional lower limb muscle strength, demonstrating improvement in both HIIT and MICT compared with the non-exercise group, but no beneficial effect of the HIIT over MICT [42]. The Herbert 6 s peak power test was used [36] to measure absolute and relative peak power of lower limb muscles; a significant increase in the HIIT compared with the control group, which remained inactive, was demonstrated. The third study assessed leg extensor muscle power [48] using the Nottingham leg extensor power rig; small beneficial effects for dominant leg power and for non-dominant leg power compared with the nonexercise group were demonstrated [48].

Two studies [43,44] measured knee extension muscle strength using an isometric/isokinetic dynamometer; HIIT did not affect the knee extension isometric and maximal concentric torque, which increased only in the RT group. 

Mass, morphology, and muscle quality (intermuscular adipose tissue and neuromuscular activation) of the quadriceps were evaluated using MRI scans and a dynamometer; it was demonstrated that HIT and RT seemed to be able to induce significant and remarkable changes in muscle mass (hypertrophy of the quadriceps muscle), morphology (increased anatomical cross-sectional area at 25%, 50%, and 75% of femur length, increased pentation angle of the fibers from the vastus lateralis) and quality (decreased intermuscular adipose tissue at 50% of femur length without additional benefit of HIIT) [43,44]. Furthermore, two positive effects (physiological cross-sectional area and voluntary activation of the quadriceps, as evaluated via the interpolated twitch technique) were demonstrated only following RT training [43,44].

Tibialis/soleus muscle activity was assessed by surface electromyography recording in two studies [33,47], including one single session of HIIT. Anterior tibialis muscle activity increased during single limb stance with eyes open (SLEO) for up to 10 min after HIIT [33]. The anterior–posterior muscle coordination pattern during single-leg stance did not change following one session of HIIT [47].

(2) Balance and subjective balance perception

(2a) Balance

Five studies assessed the effect of HIIT on balance and subjective balance perception [3,33,35,42,47]. Direct measures of static balance were performed in three studies [33,35,47] using a foot scan portable foot pressure plate [36] and piezoelectric force plate. One study reported that six weeks of HIIT once every five days did not affect static balance in double standing and in single standing [36], while the other studies demonstrated that a single session of HIIT increased postural sway immediately after HIIT during SLEO and during double-limb stance with closed eyes (DLEC) immediately and 10 min after HIIT for up to 30 min [33].

The effect of HIIT compared to the MICT group and the nonexercise group on balance was explored in one study [42] by measuring the time a participant could stand on one leg without support (one-leg standing test) in both legs. The results showed that the performance in the test on the left leg did not change in either of the groups. The performance in the right leg was significantly decreased only in the HIIT group. However, the baseline test performance was different between the groups.

The Timed Up and Go (TUG) test was used to measure dynamic balance and functional mobility in four studies [3,42,46,49]. Two of these studies demonstrated that HIIT was superior to other exercise interventions in terms of improving TUG performance compared with the MIIT group and the CON [3,49] and one study demonstrated greater improvement in TUG in the HIIT group compared with the MCT group, the RT group, or the CON [46]. In contrast, in one study HIIT and MICT had the same positive effect on TUG compared with the nonexercise group [42].

(2b) Subjective balance perception

One study [3] measured subjective balance perception/confidence via two assessment tools, the Falls Efficacy Scale (to measure fear of falling), and the Activities-specific Balance Confidence Scale (ABCS). It was shown that HIIT was more effective than MIIT and nonexercise in improving fear of falling; however, it was equal to MIIT in improving balance confidence [3].

(3) Gait and level of activity

(3a) Gait

Gait was measured in two studies (reported in three articles) [3,42,49]. One study measured the 6 min walk test (6MWT) and showed that both HIIT and MICT statistically increased gait speed compared with the nonexercise group [42]. In contrast, it was demonstrated that HIIT led to greater improvement in gait spatiotemporal parameters (using the OptoGait optical detection system) [3] and by calculating gait speed (via TUG performance) [49] compared with the MIIT and nonexercise groups. 

(3b) Level of physical activity

One study evaluated physical activity during the week by using a multi sensor activity monitor [45] an improvement in physical activity in HIIT compared with CMIT was demonstrated, as HIIT affected vigorous physical activity on training days but not on the general physical activity patterns on non-training days. HIIT did not lead to increased sedentary time as reflected in higher physical activity levels during weekdays compared with CMIT, in which physical activity during training days was increased but the overall physical activity during weekdays decreased. However, these lifestyle changes were not maintained at the two-month point after the end of the program. 

(4) Quality of life

Quality of life was evaluated in two studies [48,49]. One study [48] assessed health related quality of life using the Short Form-36 health questionnaire (SF-36). The results demonstrated that a HIIT program involving the upper- and lower-body HIIT had a small effect on the physical, general health, vitality, mental health, and bodily pain domains of the SF-36 compared to the nonexercise control group. In the second study [49], improvement of quality of life was demonstrated following HIIT program with TRX suspension training exercises compared with MIIT and nonexercise control groups. The observed improvement was in the following SF-36 domains: general health, vitality, and physical functioning [49].

##### Quality of the Study

Quality of studies detailed in Table 6.

Only 10 out of the 11 included studies could be assessed by the Pedro scale, as the study of Donath, 2015 [47] used a pre-post design and did not include a group comparison.

The PEDro scores range between 3 to 8 out of 10. Five studies were rated as high quality [3,35,42,48,49], four studies were rated as moderate quality [43,44,45,46], and one study was rated as low quality [33].

## 4. Discussion

The aim of this study was to test the effectiveness of HIIT as a treatment modality to prevent falls by targeting fall risk factors. Accordingly, we searched the literature for articles examining the effectiveness of HIIT in reducing proven major risk factors for falls, namely balance, muscle strength, and physical activity. Eleven studies involving 328 healthy older individuals were included in this review. None of the studies addressed the number of falls among the participants. The main findings of this systematic review and recommendations are discussed below.

### 4.1. HIIT Protocols Used for Older Adults

The included studies were characterized by large variations in the HIIT protocols in terms of modality, number of sessions (1–48), frequency (one session per week to three sessions per week), duration (single sessions to 18 weeks), intensity of the exercise, measure of the intensity (VO_2_ max, maximal heart rate, heart rate reserve, or rate of perceived exertion as per the Börg scale), number of bouts (4 to 12), length of activity (30 s to 4 min), and rest interval (passive recovery/active recovery with different intensity ranges between 2 to 3 min). 

A meta-analysis by Wu et. al. [27], which examined the effect of HIIT on physical fitness, metabolic parameters, and cardiorespiratory fitness in older adults determined the optimal HIIT protocol for improved VO_2_ peak (“training periods > 12 weeks, training frequencies of 2 sessions/week, session lengths of 40 min, 6 sets and repetitions, training times per repetition of >60 s, and rest times of <90 s”) [27]. However, to the best of our knowledge there is no study that reports an effective HIIT protocol for improving balance and increasing muscle strength.

There is a differentiation between a “high volume HIIT protocol” and a “low volume HIIT protocol” [51,52]. High volume is defined as intensive active intervals (i.e., not including rest periods) longer than 15 min, while low volume is defined as total active time less than 15 min [52,53]. High volume HIIT protocols included the most common HIIT protocol of 4 × 4 min, which was developed by a Norwegian research group; referred to as “Wisloff’s Group 4 × 4”, it includes four periods of four minutes of intense work at 80% to 95% of the individual’s maximum heart rate, where each period is followed by a less intensive rest period of three minutes. This HIIT protocol has provided evidence in clinical and non-clinical populations and is considered safe for patients with stable coronary heart morbidity [52,54]. Alternatively [55,56], there is no consent among researchers which of the two types of HIIT protocols is more effective in terms of cardiorespiratory fitness [32,53,55,57]. A low volume of HIIT protocol has an advantage in terms of time-saving as the training duration is shorter compared to the high volume HIIT protocol [56].

In the 11 studies included in the current study, it was not possible to point to a single protocol that was most common [3,33,43,44,47,49].

High-volume studies in the current review demonstrated better results than MCT in two studies in term of functional mobility [46] and upper limb strength (arm curl test) [42], in one of these studies [42], the effectiveness of HIIT was equal to MCIT in terms of lower limb strength. All other studies that used a high-volume HIIT protocol [3,33,47,49] did not compare HIIT with MCIT. Only one study with a low volume of HIIT used MCIT as a control group, and the results showed that HIIT is superior to MCIT in terms of level of weekly physical activity [45]. Accordingly, based on the included studies in the current systematic review, it cannot be recommended which protocol of HIIT (high or low volume) is optimal in terms of balance, muscle strength, or other fall risks.

A related point was the weekly frequency of HIIT. Sculthorpe et al. [35] studied the effect of reducing the weekly frequency of HIIT to once a week (and called it low-frequency HIIT) based on the rationale that it may be necessary to change the conventional frequency of three times per week in order to adapt the standard HIIT protocol to the unique age-related requirement of older persons, namely longer recovery time following intense exercise compared with younger individuals in order to avoid accumulated fatigue [35,58]. This rationale was reinforced by Herbert et al. [58] demonstrated that five days is the average recovery time of peak leg power output from a single session of HIIT in men aged 60 years compared with the average recovery time of three days in men aged 25 years. Sculthorpe et al. [35] demonstrated that low-frequency HIIT led to a significant improvement in peak muscle power compared with the nonexercise group. However, it is evident from only one study without blindness of the examiner using a low volume of HIIT and without comparison to the standard weekly frequency of HIIT (3 × session wk^−1^). Accordingly, further studies with high mythological quality are warranted to explore the advantage of low frequency of HIIT in elderly adults.

### 4.2. Modality

The noted heterogeneity among the studies included in this review was also expressed in the modality of the HIIT that was used. Seven of the studies included training only the lower limbs via cycling [35,43,44,45] or treadmill walking [33,46,47], and four studies included upper and lower limb exercises of different types: mesocycles (movement of the lower limbs, combined with movement of the upper limbs with or without external load [42]), upper, lower, and full body exercises using a hydraulic resistance ergometer [48], or TRX exercise [3,49].

Based on the current results, a conclusion regarding which modality is most favorable to reduce fall risk in healthy older individuals cannot be reached. Yet, previous studies have shown that the cycle ergometer was the most common type of HIIT instrument used among older persons, followed by treadmill walking [59]. Furthermore, previous reviews recommended cycling (cycle ergometer) as an effective HIIT modality to improve cardiometabolic and cardiovascular health in older persons, including additional benefits in terms of accessibility, safety, and decreased stress on joints [59,60,61].

### 4.3. Characteristic of the Participants

The maximum average age of the participants was 70 [33,47], indicating that persons older than 70 and frail older persons (older than 75) [62] were not included in the studies that examined the effect of HIIT on fall risk. Furthermore, none of the included studies considered the fall status of the participants. Accordingly, further studies should examine the effect of HIIT on specific elderly adults such as those with a history of falls or those at high risk of falling. 

Both genders were involved in six of the reviewed studies [3,33,46,47,48,49]. One study involved only women [42] and four studied involved only men [35,43,44,45]. Based on the current results, we could not differentiate the effect of HIIT on the gender of the participants.

The baseline level of activity of the participants is crucial in terms of safety issues and avoidance of adverse events [61]. Accordingly, the intensity of HIIT should be tailored to the participants’ fitness and activity baseline [61]. In addition, it has been claimed that HIIT is suitable for individuals with low levels of physical fitness who are unable to perform continuous high-intensity exercise [11].

However, in the current review, the baseline level of activity varied among the studies but can be categorized into three groups:

(1) Physically active patients with a variety of activity levels: Six studies [33,43,44,45,47,48], with only some studies reporting how activity was evaluated. The levels of activity were as follows: (a) moderately active, as assessed by the IPAQ [43,44] or by minutes of activity per day [45], and (b) active, as measured by the Freiburger Physical Activity scale [33,47]. One study [48] reported that the participants were “physically active” but had not in the previous year engaged in structured and systematic (moderate to high-intensity) endurance or strength training exercise more than twice per week.
(2) Not active: Two studies reported that the subjects were not active [35,46]. However, the definition of inactivity was completely different between the two studies [35,46]. One study defined it as no participation in any regular physical activity for a minimum of 30 years (for either recreational or work-related purposes) [35]. The other study defined it as not participating in at least 30 min of moderate-intensity physical activity (64–76% of maximal heart rate) on at least three days of the week for the previous three months [63].
(3) The level of activity of the participants was not reported [3,42,49]. Based on the current review, it is not clear whether the base level of activity of older adults is a contributing factor to the effect of HIIT on fall risk, as was demonstrated in a previous meta-analysis which showed that the positive effect of HIIT on the VO_2_ max values in healthy, young to middle-aged adults was higher in the less fit participants [64]. Further studies should examine the effect of HIIT on fall risk factors in elderly adults engaged in different levels of activity.

### 4.4. Effect of HIIT on Level of Balance and Postural Control

Balance and postural control are crucial factors that affect the incidence of falls in older adults [65,66]; accordingly, we examined the effect of HIIT on balance in older persons. The included studies explored the effect of HIIT on balance by examining static or dynamic balance [3,33,35,42,46,49]. The effect of HIIT on static balance could not be determined because of insufficient data as only two studies [33,35] investigated this issue and they were incompatible. One of the two studies [36] examined the effect of six weeks of HIIT, while the second study [33] examined the effect of a single HIIT session with results being completely diverse. While six weeks of HIIT demonstrated no effect on static stability [35], the second study that used a single session of 4 × 4 min at 90% of HR max walking on a treadmill increased postural sway during double-limb support compared with no application of HIIT immediately and 10 min after the end of the training, even up to 30 min until returning to baseline in the case of occluded vision in healthy older adults [33]. Based on this finding, we recommend that clinicians and trainers be cautious during the initial stage of an HIIT program, especially in older subjects with a sedentary lifestyle. Accordingly, older persons should be monitored and supervised because of the fear of the negative consequence of continuous instability (10 to 30 min) following a single HIIT session. The authors of this study [33] referred to this phenomenon as “an acute open-fall-window” indicating that a single session may be dangerous to older persons as it may cause fall due to reduce stability. On the basis of the findings of the second study [35] that involved a prolonged HIIT program over several weeks, it is not possible to answer the question raised by the Donath et al. study [33] regarding whether the acute negative effect of a single HIIT application on static balance will diminish (adaptation) over prolonged repeated sessions of HIIT as the two studies [33,35] are not comparable in terms of the HIIT protocol (such as treatment modality, dose of the high-intensity, and active recovery). Furthermore, the prolonged HIIT program was conducted in a lower total volume (total of six sessions) and lower frequency of weekly HIIT sessions that is lower than the commonly used protocol of HIIT, which is composed usually of three weekly sessions [34].

The TUG test is one of the most commonly used tools for measuring functional balance in fall risk assessment and is recommended by the American Geriatrics Society and the British Geriatric Society as an assessment tool for fall risk [67]. In accordance with the goal of this systematic review, it is crucial to explore the effect of HIIT on TUG performance in older persons. The current systematic review revealed that the TUG test was measured in three studies (four papers) [3,42,46,49], all of which demonstrated improvement in the TUG test following HIIT. No consistent results were found regarding the effect of TUG compared with other treatment modalities, with one study demonstrating equal improvement in TUG following HIIT and moderate continuous training compared with the nonexercise group [42]. In contrast, two other studies demonstrated the superiority of HIIT in improving TUG performance compared with MCT [46], RT [46] or MIIT [3,49] to the nonexercise group [3,46,49]. It is possible that the noted improvement in the TUG test is a result of improvement in dynamic balance as well as in the strength of the lower limb muscles due to the HIIT [68]. However, these studies [3,42,46,49] are not comparable due to different HIIT protocols in terms of modality and the dose of the treatment.

As mentioned, the current review included only older individuals without balance impairments. However, there is evidence for the potential of HIIT in older persons with balance impairments; for example, a systematic review reported that HIIT caused significant improvements in the TUG test and the Berg Balance Scale (BBS) among post-stroke patients [69]. Similarly, an RCT demonstrated that a six-week HIIT program improved dynamic balance evaluated by the Y Balance Test in subjects with type 1 diabetes mellitus [70].

An interesting finding that was explored in the current review was that fear of falling improved more following HIIT than after MIIT [3]. This finding has a clinically important implication as the rule of fear of falling in terms of fall prevention has recently received greater attention because the subjective psychological aspect of fear and anxiety should be considered as a fall risk [71]. Similarly, this study [3] demonstrated that HIIT improved the participants’ confidence in their ability to perform daily life activity tasks without losing balance and becoming unsteady, as assessed by the ABCS compared with MIIT (HIIT: difference = 1.12; MIIT: difference = 0.63); however, the difference did not reach statistical significance.

### 4.5. Effect of HIIT on Lower Limb Muscle Strength and Activity

HIIT was demonstrated in two studies as an effective modality to improve the strength of the lower limbs as reflected in improved performance of the Herbert 6-s peak power test compared with the nonexercise regimen [35] and in improved performance of 30 s sit to stand test, which was equal to the improvement with MCT [42]. Similar results were found in individuals with Parkinson’s disease, in which HIIT and moderate-intensity continuous exercise training improved the performance of the five times sit to stand test [72].

The effect of HIIT on quadriceps muscle strength was tested in only three papers [43,44,48] with contrasting results. HIIT demonstrated no effect on quadriceps strength compared with RT [43,44]. In contrast, the second study [48] demonstrated that HIIT had a mild possible effect on muscle strength compared with the nonexercise regimen. It is difficult to compare the two studies because they used different HIIT protocols [43,44,48].

HIIT was also found to have no effect on the activity of the tibialis/soleus muscles during single-limb leg support [33,47].

The current results contradict the results of a recent meta-analysis that concluded that muscle strength was significantly higher in older adults after HIIT interventions [27]. However, it should be emphasized that the meta-analysis’s [27] conclusion was based on data from five studies, two of which examined upper limb muscles and three lower limb muscles. Of the latter three studies, one did not apply HIIT but vigorous exercise intensity [73], and another included obese older adults who were divided according to daily protein intake distribution [74]. Both of these studies were not included in the current review as they did not meet the inclusion criteria. The third study was included in the current review [48].

Accordingly, based on the findings of the current review and the existing knowledge in the literature on HIIT in general, we believe that the effect of HIIT on the quadriceps or other specific lower limb muscle group strength is not clearly demonstrated in healthy older individuals. 

### 4.6. Effect of HIIT on Gait and Level of Activity

HIIT was found to improve the gait performance of healthy older persons in terms of six MWT performance compared with the nonexercise group and was similar to the effect of MICT [42]. In addition, HIIT demonstrated greater improvement in gait spatiotemporal parameters compared with MIIT and the nonexercise group [3,49]. These two studies are not compatible because of different HIIT protocols [3,42,49]. The increase in gait performance may be due to an increase in cardiorespiratory fitness following HIIT [75].

An interesting point that emerged in one study [45] was that HIIT did not increase the sedentary time (measured by the amount of weekly hours of physical activity) in non-training healthy older persons compared with CMIT.

### 4.7. Effect on HIIT on Quality of Life

HIIT was found to have a positive effect on quality of life in healthy older persons, as evaluated by the SF-36 questionnaire, compared with MIIT [49] or the nonexercise group [48]. The two studies [48,49] are not comparable in terms of the HIIT protocol and the magnitude of the resulting effect.

These results demonstrate that the positive effect of HIIT on quality of life is consistent with previous results in adults and older subjects with and without morbidity [32,76,77].

### 4.8. Safety, Feasibility and Responsiveness of HIIT

HIIT training seems to be a safe training modality for the older adult population, as none of the included studies reported any adverse effects [3,33,35,42,43,44,45,46,47,48,49].

A clear conclusion regarding the dropout rate and level of attendance compared with other training modalities cannot be made based on the included studies, as there is not enough data, and the reported data are incomplete in some of the studies. Furthermore, the reported advantage of higher participants responsiveness to HIIT compared with MCT [30] was not demonstrated in the current review. One study demonstrated lower dropout rates in the HIIT group compared with the MCIT group (HIIT = one participant versus MCT = six participants) [42], while another study [46] reported a higher dropout rate in the HIIT group compared with the MCT group (HIIT = 11 participants versus MCT = zero participants). However, the two studies were not comparable because of the heterogeneity of the training protocols. However, based on the available data from the included studies [3,35,42,43,44,45,48,49], it seems that HIIT was well attended by older participants.

It should be noted that the HIIT was performed under supervision and in a clinical setting to ensure the maintenance of the study protocol and the safety of the participants (maintaining the required balance between high intensity of heart rate for a fixed short time along with sufficient recovery time between bouts). However, such a training setting raises the question of the applicability of HIIT for independent performance by older persons at home. Moreover, none of the included studies tested the therapeutic effects of HIIT over time. This also calls into question the degree of applicability of the HIIT program over time, even in a clinical setting.

Only one study [48] examined the feasibility of performing the HIIT program in a group setting, which has some benefits such as increased compliance, decreased dropout level, social interaction between the participants, and streamlining financial resources and manpower [78].

### 4.9. Strength and Limitations

This is the first study in which the effects of HIIT on fall risk and balance in healthy older individuals were systemically pooled based on a literature review. In the present systematic review, it is impossible to determine the influence of HIIT due to the small number of studies and the high degree of heterogeneity. The generalizability and transferability of the clinical recommendations is limited as the studies were performed in older adults without balance impairments and not in the fall-prone population.

## 5. Conclusions

The systematic review of the literature yielded only 11 papers (9 studies) that met the inclusion criteria. The included studies were characterized by high heterogeneity in terms of methodology, HIIT modality, HIIT protocol, patient characteristics, comparison group, and outcome measures. Since there is insufficient data and no consensus among the trials regarding the effect of HIIT, it is difficult to conclude whether HIIT is an effective method for reducing fall risk or improving balance in healthy older adults. Further research is needed to explore the effect of the HIIT protocol as a fall prevention modality for older adults compared with other treatment modalities, including determining the optimal HIIT protocol to achieve this target. However, based on the currently available evidence regarding HIIT, it cannot be recommended as a single modality for fall prevention strategies in individual seniors living in the community. However, HIIT may be considered as a supplement to proven fall prevention programs such as the Otago exercise program [79] because of the potential of HIIT to improve functional lower limb strength reflected in functional performance tests (such as the sit-to-stand test). HIIT also has the potential to improve dynamic balance (TUG test performance) and subjective balance perception. HIIT seems to be a safe and well-tolerated modality in an older population. However, caution is warranted following HIIT, especially after the first session, due to possible temporary instability. Older individuals should be warned that they may suffer from temporary instability that will last up to half an hour. Therefore, attention is needed in this period of time while performing unstable physical activity (such as during a shower or using an escalator).

## Figures and Tables

**Table 1:** Characteristics of the participants.

	Study	Place	Age *M* (*SD*) Years	Number of Participants (Total and in Each Group)	Gender of the HIIT Group M/F (Number)	Baseline Activity Level	Enrollment Prerequisite
1	Bruseghini, 2015 [43]	Italy	68 (4)	12	12/0	Moderately active.	Medical examination, preliminary cycle-ergometer stress test.
2	Bruseghini, 2019 [44]	Italy	69.3 (4.2)	12	12/0	Moderately active- IPAQ score: 4333 ± 1750, MET min week^−1^.	IPAQ, medical examination.
3	Donath, 2015 [33]	Germany	70.0 (3.8)	40 20 active older adults. 20 young adults. Age: 27.1(3) years.	8/12	Active older adults—Freiburger Physical Activity: 10.9(5.8) h·week.	Medical examination, PAR-Q, resting ECG in supine and exercise ECG and maximal heart rate in exhaustive ramp-like treadmill exercise testing
4	Donath, 2015 [47]	Germany	70.0 (3.8)	40 20 active older adults. 20 young adults. Age—(27.1) (3)	8/12	Active older adults—Freiburger Physical Activity: (10.9) (5). 8 h·week.	Not reported.
5	Coetsee, 2017 [46]	South Africa	All groups—only range reported (55–75). HIIT group—64.5(6.3). RT group—62.4(5.1). MCT group—61.6(5.8). CON group—62.5(5.6).	77 4 groups: HIIT—13 RT—22 MCT—13 CON—19	3/10	Inactive—not been participating in at least 30 min of moderate-intensity physical activity (64–76% of maximal heart rate) on at least 3 days of the week for the previous 3 months. No report how this information was collected.	Screening procedure to identify eligibility—ECG in rest, waist-to-hip ratio, BMI, MoCA, TUG.
6	Sculthorpe, 2017 [36]	West Scotland	All groups—Limited age group of 56–65 years old. HIIT—62.3(4.1). CON—61.6(5.0).	33 HIIT—22 CON—11	22/0	Lifelong sedentary—self report of not being involved in any regular and formal physical activity for either recreational or work-related purpose for minimum of 30 years.	General medical practitioners to take part in strenuous physical activity. Completion of the PAR-Q. Cardiorespiratory fitness was established in an exercise physiology laboratory by indirect calorimetry.
7	Ballesta-García, 2019 [42]	Spain	All groups—67.8(6.2). Age of each group—not reported.	54 HIIT—18 MICT—18 CON—18	0/54	Independent. In activities of daily living—Lawton and Brody and Katz scale and PAR-Q negative or only item 6 positive and controlled.	General medical evaluation to ensure physically and mentally ability to perform in the intervention. Excluded those participating in or who had previously participated in a similar exercise program in the past three months.
8	Hurst, 2019 [48]	United Kingdom	HIIT—range: 50–81, average: 61.9, *SD* not reported. CON—range: 50–74, average: 62.8. *SD* not reported.	36 HIIT—18 CON group—18	11/7	Physically active—not engaged in structured and systematic (moderate to high-intensity) endurance or strength training exercise more than twice per week in the previous year.	Medical screening questionnaire.
9	Jiménez-García, 2019 [3]	Spain	All groups (all of whom completed the intervention)—68.49 (5.18). HIIT—68.23 (2.97). MIIT—68.75 (5.98). CON—68.52 (6.33).	82 reduced to 73. HIIT—28 reduced to 26. MIIT—27 reduced to 24. CON—27 reduced to 23.	2/24	Not reported.	Being over 60 years old, not suffering from conditions that were contraindicated in exercise program. Excluded those who were already included in other training program.
10	Jiménez-García, 2019 [49]	Spain	All groups (all of whom completed the intervention)—68.49 (5.18). HIIT—68.23 (2.97). MIIT—68.75 (5.98). CON—68.52 (6.33).	82 reduced to 73. HIIT—28 reduced to 26. MIIT—27 reduced to 24. CON—27 reduced to 23.	2/24	Not reported.	Being over 60 years old, not suffering from conditions that were contraindicated in exercise program. Excluded those who were already included in other training program.
11	Bruseghini, 2020 [45]	Italy	All groups—not reported. HIIT—69.4(4.3). MCIT—69.7(4.1).	24 HIIT—12 MCIT—12	24/0	Moderate physical activity > 90 min/d.	A preliminary medical examination with a cycle ergometer stress test to exclude abnormal response to exercise. Requirement of normal ECG at rest.

*SD*, standard deviation; *M*, male; F, female; IPAQ, International Physical Activity Questionnaire; MET, average daily metabolic equivalent; h, hours; ECG, electrocardiogram; HIIT, high-intensity interval training; RT, resistance training; MCT, moderate continuous aerobic training; CON, control; BMI, Body Mass Index; MoCA, Montreal Cognitive Assessment; TUG, Timed Up and Go test; s, second; MICT, moderate-intensity continuous training; PAR-Q, Physical Activity Readiness Questionnaire; MIIT, moderate-intensity interval training; MCIT, moderate continuous intensity training.

**Table 2:** Characteristics of the HIIT protocols.

	Study Author, Year	HIIT Group					
		Duration/Frequency (Per Week)/Total Amount	Mode	Nu. Bouts	Work Intensity	Rest Intensity	Work & Recovery (Rest) & Total Duration
1	Bruseghini, 2015 [43]	8 w/(3)/24	Cycling exercise	7	85–95% VO_2_ max	40% VO_2_ max	2 min work, 2 min active recovery, total 55–60 min included warm-up and cooldown phase.
2	Bruseghini, 2019 [44]	8 w/(3)/24	Cycling exercise	7	85–95% VO_2_ max	40% VO_2_ max	2 min work, 2 min active recovery, total 55–60 min included warm-up and cooldown phase.
3	Donath, 2015 [33]	Single session	Treadmill walking ^$^	4	90–95% HR max	70% HR max	4 min work, 3 min active recovery, total not reported, assumed to be ≈28 min.
4	Donath, 2015 [47]	Single session	Treadmill walking ^$^	4	90–95% HR max	70% HR max	4 min work, 3 min active recovery, total not reported, assumed to be ≈28 min.
5	Coetsee, 2017 [46]	16 w/(3)/48	Treadmill walking ^$^	4	90–95% HR max	70% HR max	4 min work, 3 min active recovery, total ≈ 30 min, warm-up and cooldown phase not reported.
6	Sculthorpe, 2017 [36]	6 w/1 every 5 days/9	Cycle ergometer	6	>90% HRR was measured as 50% PP in the last 6 (out of 9) sessions while in the first 3 sessions it was 40% PP	Not reported	30 s work, 3 mins active recovery, total assumed to be ≈ 21 min, only states 5 min of warm-up and no details as to the duration of the cooldown phase.
7	Ballesta-García, 2019 [42]	18 w/(2)/36	Mesocycles -movement of the lower and upper limbs with/without external load	6–8 to 8–12	14–15 RPE to 16–18 RPE. Gradually increased every 3 w out of the 18 w	From 7–8 RPE to 10–11 RPE. Gradually increase each 3 w out of the 18 w	1–1.5 min work, 2–2.5 min active recovery, total- from 18–32 to 28–40 min during the last 3 w out of 18 of the intervention
8	Hurst, 2019 [48]	12 w/(2)/24	Upper, lower, and full body exercises using a hydraulic resistance ergometer	4	90% of HR max	Passive recovery	Started at 45 s, increased by 10 s at the end of every 3rd w (out of 12) to 1.25 min (by w 10), 3 min passive recovery remained constant over the course of the intervention, total increasing from 12 to 20 min. Each session included ≈6 min of warm-up phase & ≈4 min of cooldown phase.
9	Jiménez-García, 2019 [3]	12w/(2)/24	Suspension training system (TRX)	4	90–95% HR max	50–70% HR max	4 min work, 3 min active recovery, total ~28 min. 10 min warm-up phase & 10 min cooldown period.
10	Jiménez-García, 2019 [46]	12w/(2)/24	TRX	4	90–95% HR max	50–70% HR max	4 min work, 3 min active recovery, total ~28 min. 10 min warm-up phase & 10 min cooldown period.
11	Bruseghini, 2020 [45]		Cycling exercise	7	85–95% VO_2_ max	40% VO_2_ max	2 min work, 2 min active recovery, –total—45 to 60 min including 15 min warm-up phase.

Nu, number; w, weeks; VO_2_, maximal oxygen consumption; min, minute; HR, heart rate; max, maximum; ≈, approximately; HRR, heart rate reserve; PP, peak power; RPE, rate of perceived exertion as per Börg scale; TRX, suspension weight training. ^$^, treadmill walking with speed and inclination adjustments when needed to maintain the targeted intensity.

**Table 3:** The characteristics of the control groups in the studies under review.

	Study Author, Year	Intervention Group			Control Group Non-Exercise Group (Yes/No)
		Modality	Duration/Frequency (Per Week)/Total Amount	Intensity	
1	Bruseghini, 2015 [43]	RT—Bilateral resistance exercise using leg press flywheel ergometer	8 w/3/24	4 sets of 7 maximal bilateral knee concentric extensions and eccentric flexions of the knee from about 90° to 160–170° knee joint interspersed by 3 min rest periods were initiated immediately following two submaximal actions. 10 min warm-up including 3 sets of 7 submaximal actions with progressively increased effort. Session duration 15 min including warm-up and rest periods.	No.
2	Bruseghini, 2019 [44]	The same as paper number 1			
3	Donath, 2015 [33]	Treadmill walking	Single session	Comfortable normal walking speed below 50% of HR max for 4 × 4 min. During the 3 min breaks, participants stood still in an upright position.	No.
4	Donath, 2015 [47]	Not relevant, pre-post design			
5	Coetsee, 2017 [46]	1. RT group. 2. MCT group—walking on treadmill.	16 w/3/48	1. RT group—upper and lower body resistance exercises using machines and free weights. Three sets of 10 repetitions were performed at 50%, 75%, and 100% of the individual’s 10 RM. After 8 weeks the load for each set was increased to 75%, 85%, and 100% of the individual’s 10 RM. Duration of the RT session was approximately 30 min, excluding the warm-up and cooldown. 2. MCT group performed continuous walking on a treadmill at 70–75% of maximal HR. Duration—47 min.	Yes.
6	Sculthorpe, 2017 [36]	No			Yes.
7	Ballesta-García, 2019 [42]	MCT	2 w/18/36	Similar to HIIT movements of the lower limbs & upper limbs with or without external load. 9–14 perceived exertion score as per the Börg scale. Duration—1 h including warm-up and cooldown phase.	Yes.
8	Hurst, 2019 [48]	No			Yes.
9	Jiménez-García, 2019 [3]	MIIT	12 w/2/24	Same protocol as HIIT with lower intensities: 70% of the maximum HR for the main squat activity with TRX and 50–55% of the maximum HR for the active rest intervals.	Yes—2 × 90 min health education classes focused on health promotion during the study period.
10	Jiménez-García, 2019 [49]	MIIT	12 w/2/24	Same as paper 9.	Yes, yet it was reported that the participants were instructed to maintain their daily lifestyle including guidelines to encourage physical activity but were instructed to refrain from participating in any systematized exercise activity.
11	Bruseghini, 2020 [45]	MCT	8 w/3/24	Stationary bike cycling or treadmill walking at 46–64% of VO_2_ max. Duration—20–30 min.	No.

RT, resistance training; w, weeks; HR, heart rate; MCT, moderate continuous aerobic training; RM, repetition maximum; HIIT, high-intensity interval training; MIIT, moderate intensity interval training; TRX, suspension weight training; VO_2_, maximal oxygen consumption.

**Table 4:** Number of dropouts and percentage of attendance for each study group.

	Study Author, Year	Number of Dropouts/Percentage of Attendance for Each Group
1	Bruseghini, 2015 [43]	0/100%
2	Bruseghini, 2019 [44]	0/100%
3	Donath, 2015 [33]	Older adults—0/NR Young adults—0/NR
4	Donath, 2015 [47]	Older adults—0/NR Young adults—0/NR
5	Coetsee, 2017 [46]	HIIT group—2/86.67% RT group—2/91.67% MCT group—0/100% CON group—3/86.36%
6	Sculthorpe, 2017 [36]	0/all participants completed at least 80% of training sessions
7	Ballesta-García, 2019 [42]	HIIT group—1/94.44% MICT group—6/86.67% CON group—6/86.67%
8	Hurst, 2019 [48]	HIIT group—0/99% (429/432); “42 individual sessions rearranged (≈10%) throughout the intervention period to offset participant unavailability and maximize attendance.” CON group—0/Sixteen participants completed all 24 HIIT sessions, one participant completed 23 sessions and one participant completed 22. Participants were required to attend a minimum of 90% (≥22/24) of the sessions
9	Jiménez-García, 2019 [3]	HIIT group—2/92.86% MIIT group—3/88.89% CON group—4/85.19%
10	Jiménez-García,2019 [49]	HIIT group—2/92.86% MIIT group—3/88.89% CON group—4/85.19%
11	Bruseghini, 2020 [45]	HIIT group—0/100% CON group—0/100%

NR, not reported; HIIT, high-intensity interval training; RT, resistance training; MCT, moderate continuous aerobic training; CON, control group; HIIT, high-intensity interval training; MICT, moderate-intensity continuous training; MIIT, moderate-intensity interval training.

**Table 5:** Summary of outcome measures, study design, measurement, and results.

	Study Author, Year	Domain, Measurement	Study Design	Number/Time of Measurement	Results
1	Bruseghini, 2015 [43]	Lower limb strength, muscle area, volume and activity MRI scan & DXA.	Within-subject design	2/Pre & post intervention. Fixed sequence (HIIT, 4 months of detraining followed by RT).	Hypertrophy of the quadriceps muscle in HIIT & RT. Maximal voluntary isometric torque & the isokinetic concentric-eccentric torque of quad. Increase only after RT.
2	Bruseghini, 2019 [44]	Lower limb strength, mass, morphology & quality isometric/isokinetic dynamometer	Within-subject design	2/pre- and postintervention. Fixed sequence (HIIT, 4 months of detraining followed by RT).	Knee extension isometric—increased only in RT. Maximal concentric torque—increased only in RT. Anatomical cross-sectional area at 25%, 50%, and 75% of femur length- increased in HIIT and RT. Physiological cross-sectional area—increased only in RT. Quad volume—increased in HIT and RT. Intermuscular adipose tissue—at 50% of femur length decreased after both HIT and RT in particular after RT. Pennation angle (θp) of the fibers from the vastus lateralis—increased both after HIIT and RT. Voluntary activation of the Quad (%Act)—increased only in RT.
3	Donath, 2015 [33]	Balance—piezoelectric force plate. Muscle activity of lower limbs—surface electromyography recording.	Crossover design	5/Pre HIIT, immediate post HIIT, post 10 min, post 30 min, and post 45 min	Postural sway—increases immediately after HIIT during SLEO. Increased sway during DLEC immediately and 10 min after HIIT up to 30 min. Muscle activity increased during SLEO for anterior tibialis until 10 min post HIIT.
4	Donath, 2015 [47]	Balance—piezoelectric force plate. Muscle activity of lower limb—surface electromyography recording.	Pre-post design. Comparison with young adults.	2/Pre and post single session of HIIT	No change in the ankle muscle coordination patterns during DLEC and SLEO. Pattern of elevated postural sway only during SLEO due to higher relative contribution of the tibialis muscle was not changed post HIIT. Pattern of co-activation of higher tibialis/soleus muscle activity only in SLEO was not affected by HIIT.
5	Coetsee, 2017 [46]	Physical function—TUG	RCT	2/Pre and post intervention groups	TUG HIIT > MCT and RT and CON
6	Sculthorpe, 2017 [36]	Balance muscle—Footscan portable foot pressure plate. Lower limb strength—Herbert 6-s peak power test.	RCT	3/On enrolment to the study, after conditioning exercise and after the HIIT	Static balance in double standing and while single standing—no effect of HIIT. Peak power output relative to total body mass to fat-free mass—increased only in the HIIT compared with non exercise group.
7	Ballesta-García, 2019 [42]	Upper limb strength—30-s arm curl test & maximal handgrip strength. Lower limb strength—30 s sit-to-stand (STS-30). Mobility— TUG & 6MWT. Balance—one-leg standing test.	RCT	2—pre and post intervention group	Arm curl test—HIIT > MICT and CON. Lower limb strength & TUG&6MWT HIIT & MICT > CON. One-leg standing test—left leg: no change in both groups; right leg: decreased only in HIIT group.
8	Hurst, 2019 [48]	Leg extensor muscle strength—Nottingham leg extensor power rig. Handgrip strength—dynamometer. Health-related quality of life—SF-36.	RCT	2-pre-post-intervention (~3–7 days following final training session)	* HIIT showed possibly small beneficial effects for dominant leg power, non-dominant leg power and non-dominant handgrip strength compared to CON. Trivial effect for the dominant handgrip strength. Possibly small beneficial effects for role-physical, general health, vitality, and mental health of the SF-36. Likely small beneficial effect for bodily pain in the HIT group compared with CON. Possibly moderate beneficial effect for role emotional.
9	Jiménez-García, 2019 [3]	Balance confidence— 1. Activities-specific Balance Confidence Scale. 2. Fear of falling -Falls Efficacy Scale. Dynamic balance—TUG. Gait analysis—OptoGait optical detection system.	RCT	2/-pre-intervention and post-intervention	Balance confidence—HIIT & MIIT > CG. Fear of falling, gait analysis, & TUG— HIIT > MIIT & CON.
10	Jiménez-García, 2019 [49]	Handgrip strength—dynamometer. Functional mobility and balance— 1.TUG. 2.Gait speed—estimated by the formula [6/(TUG time) * 1.62]. Health-related quality of life—SF-36.	RCT	2-pre-intervention and post-intervention	Handgrip strength increase was also observed after HIIT, but no differences were observed with MIIT and CG. Gait speed & TUG—HIIT > MIIT & CON. SF-36 domains: general health, vitality & physical functioning HIIT > MIIT & CON.
11	Bruseghini, 2020 [45]	Level of physical activity—multisensor activity monitor	RCT	3/-worn for 1 week during the 2 months before the start of training (T1), 1 week during training (T2) (randomly during weeks 5, 6, and 7 of training), and then for 1 week during 2 months after the end of training (T3)	HIIT affected vigorous physical activity on training days but not on the general physical activity patterns on non-training days. HIIT increase in levels of physical activity these lifestyle changes were not maintained at 2 months after the end of the program. HIIT does not increase sedentary time. Higher physical activity levels during weekdays were found in the HIIT group compared with CMIT. CMIT group increased physical activity during training days but decreased the overall physical activity during weekdays.

Only outcome measures that fit the inclusion criteria were reported. Only results with statistical significance were reported. MRI, magnetic resonance imaging; HIIT, high-intensity interval training; RT, resistance training; Quad, quadriceps muscle; Act, activation; SLEO, single limb stance with eyes open; DLEC, double-limb stance with closed eyes; RCT, randomized control trial; MCT, moderate continuous aerobic training; CON, control; TUG, Timed Up and Go test; MICT, moderate-intensity continuous training; MIIT, moderate-intensity interval training; 6 MWT, 6-min walk test, SF-36, Short Form-36 health questionnaire. * The effect was evaluated by calculating the mean intervention effect for each outcome, together with the confidence interval (uncertainty) classified as three levels of probability of the true effect, that is, trivial, beneficial, or harmful and defined as most unlikely or almost certainly not (<0.5%), very unlikely (0.5–5%), unlikely or probably not (5–25%), possibly (25–75%), likely (75–95%), very likely (95–99.5%), most likely (>99.5%) [48].

**Table 6:** PEDro scale of the included studies.

	Study Author, Year	1	2	3	4	5	6	7	8	9	10	11	Total (/10)	Quality
1	Bruseghini, 2015 [43]	Y	N	N	Y	N	N	N	Y	Y	Y	Y	5	Moderate
2	Bruseghini, 2019 [44]	Y	N	N	Y	N	N	N	Y	Y	Y	Y	5	Moderate
3	Donath, 2015 [33] *	N	Y	N	N	N	N	N	N	N	Y	Y	3	Low
4	Coetsee, 2017 [46] *	Y	Y	N	Y	N	N	N	Y	N	Y	Y	5	Moderate
6	Sculthorpe, 2017 [33] *	N	Y	N	Y	N	N	N	Y	Y	Y	Y	6	High
7	Ballesta-García,2019 [42] *	Y	Y	Y	Y	N	N	Y	N	Y	Y	Y	7	High
8	Hurst,2019 [48]	Y	Y	Y	N	N	Y	Y	Y	Y	Y	Y	8	High
9	Jiménez-García,2019 [3] *	Y	Y	Y	Y	N	N	N	Y	N	Y	Y	6	High
10	Jiménez-García,2019 [49] *	Y	Y	Y	Y	N	N	N	Y	N	Y	Y	6	High
11	Bruseghini, 2020 [45]	Y	Y	N	Y	N	N	N	N	N	Y	Y	4	Moderate

1. Eligibility criteria; 2. Random allocation; 3. Concealed allocation; 4. Baseline comparability; 5. Blind subjects; 6. Blind therapists; 7. Blind assessors; 8. Outcomes were obtained in more than 85% of the subjects; 9. Intention-to-treat analysis; 10. Between-group comparisons; 11. Point estimates and variability, the eligibility criteria did not add to the total score. Y, Yes; N, No. * Appears in the PEDro database.
